# Impact of Smoking on Outcomes in HPV-Positive Oropharyngeal Squamous Cell Carcinoma in a Chinese Cohort Under AJCC 8th Edition Staging

**DOI:** 10.3390/jcm14196802

**Published:** 2025-09-26

**Authors:** Yingying Zhu, Wenwen Diao, Xiaoli Zhu, Shuting Yu, Xin Xia, Wei Han, Xingming Chen

**Affiliations:** 1 Department of Otolaryngology Head and Neck Surgery, Peking Union Medical College Hospital, Peking Union Medical College and Chinese Academy of Medical Sciences, No 1 Shuaifuyuan, Wangfujing, Beijing 100730, China; zhuyingying10690@pumch.cn (Y.Z.); diaowenwen1@pumch.cn (W.D.);; 2 Department of Epidemiology and Statistics, Institute of Basic Medical Sciences, Chinese Academy of Medical Sciences & School of Basic Medicine, Peking Union Medical College, No. 5, Dongdan Third Road, Beijing 100730, China

**Keywords:** human papillomavirus (HPV), oropharyngeal squamous cell carcinoma (OPSCC), smoking history, prognosis, AJCC 8th edition staging

## Abstract

**Objectives**: Human papillomavirus (HPV)-positive and HPV-negative oropharyngeal squamous cell carcinoma (OPSCC) represent biologically distinct subtypes. However, the role of tobacco exposure in the pathogenesis of each remains incompletely understood. This study aimed to evaluate the prognostic implications of smoking in patients with HPV-positive OPSCC, with stratification based on the eighth edition of the American Joint Committee on Cancer (AJCC-8) staging system. **Methods**: We retrospectively analyzed all OPSCC cases managed at our institution between January 2011 and January 2024. Smoking history was dichotomized into <10 and ≥10 pack-years. Survival outcomes—including overall survival (OS), disease-specific survival (DSS), and progression-free survival (PFS)—were calculated using the Kaplan–Meier method. Log-rank testing and multivariable Cox proportional hazards modeling were used to assess prognostic factors and identify risk groups. An interaction analysis was also conducted to determine whether smoking alters the survival benefit conferred by HPV positivity. **Results**: Of the 329 patients included, 181 (55%) had a history of smoking, while 148 (45%) had never smoked. Among all patients, 211 (64.1%) were HPV-positive. HPV-positive cases exhibited superior 3- and 5-year OS, DSS, and PFS compared with HPV-negative tumors (*p* < 0.001). Within the HPV-positive cohort, never-smokers had the most favorable survival outcomes. Notably, interaction modeling demonstrated that the survival benefit of HPV positivity was markedly diminished among smokers, with hazard ratios approaching unity. **Conclusions**: Tobacco use negates the survival advantage typically associated with HPV-positive OPSCC. These findings highlight the critical need to account for smoking history in treatment planning and when considering eligibility for de-intensification strategies in HPV-related diseases.

## 1. Introduction

Human papillomavirus (HPV) has been firmly identified as a key etiological agent in oropharyngeal squamous cell carcinoma (OPSCC), particularly in tumors arising from the tonsils and base of the tongue [1,2,3]. This shift in disease epidemiology has led to substantial improvements in treatment outcomes, including disease-specific and overall survival (OS) rates [4,5].

HPV-related OPSCC is now regarded as a biologically distinct subset of head and neck squamous cell carcinoma (HNSCC), differing significantly from its HPV-negative counterpart in terms of epidemiologic trends, risk profiles, therapeutic strategies, and clinical prognosis [6,7,8,9]. Reflecting this distinction, the most recent (eighth) edition of the American Joint Committee on Cancer (AJCC-8) staging system incorporates HPV status into the staging criteria for OPSCC [10]. In parallel, the National Comprehensive Cancer Network (NCCN) promptly updated its clinical guidelines, aligning with the revised TNM staging for HPV-associated OPSCC and establishing separate diagnostic and therapeutic recommendations [11].

Nevertheless, classifying OPSCC solely on the basis of HPV status overlooks the prognostic significance of coexisting risk factors, particularly smoking. The potential interactions between smoking and HPV-positive OPSCC are especially relevant in the context of ongoing efforts to de-escalate therapy for HPV-driven cancers [12]. Without accounting for behavioral or comorbid risk modifiers such as tobacco use, treatment deintensification may result in unintended negative outcomes for high-risk individuals [13].

While the AJCC-8 staging system has been validated across several patient cohorts [14,15,16,17,18,19,20], most epidemiological investigations of HPV-positive OPSCC originate from Western populations. Consequently, their findings may not be applicable to regions with differing cultural and behavioral practices that may influence disease etiology [21]. Broader international studies are needed to develop tailored clinical approaches and inform regional public health strategies.

This study aimed to evaluate the prognostic value of smoking among patients with HPV-positive OPSCC using data from a single institutional database. Specifically, we sought to determine the impact of tobacco exposure on survival outcomes while adjusting for the AJCC-8 stage. We hypothesized that smoking would serve as a significant prognostic factor even within the context of the new staging framework. Crucially, the specific impact of smoking on survival outcomes within the context of the AJCC-8 staging system, particularly within an Asian population where behavioral patterns and potential genetic factors may differ, remains inadequately explored. This study addresses this significant knowledge gap.

## 2. Materials and Methods

### 2.1. Study Design and Population

A retrospective review was conducted of all patients diagnosed with oropharyngeal squamous cell carcinoma (OPSCC) and treated at Peking Union Medical College Hospital (PUMCH) between January 2011 and January 2024. The study protocol received approval from the PUMCH Ethics Committee (code: I-23PJ073; date: 10 January 2023). Histopathological diagnoses were made using specimens obtained through surgical resection or biopsy, with confirmation provided by a board-certified pathologist. All tissue samples were sourced from the Clinical Biobank (ISO 20387 [22]) at PUMCH, affiliated with the Chinese Academy of Medical Sciences.

Patients were excluded if they had a second primary malignancy; if their primary tumor arose outside the oropharynx (e.g., oral cavity, larynx, or hypopharynx); if their diagnosis was not squamous cell carcinoma; if they had previously received palliative care; or if tissue samples or follow-up data were incomplete.

### 2.2. Demographic and Clinical Data

Patient demographics—such as age at diagnosis, sex, tobacco and alcohol use—along with clinical variables—including HPV status (determined by p16 immunohistochemistry or HPV-DNA testing of tumor samples), treatment modality, presence of regional or distant metastasis, and survival status—were obtained from the institutional electronic medical records.

Smoking status was defined based on a threshold of more than 10 pack-years, consistent with prior studies identifying this cutoff as a significant survival predictor [23].

Alcohol consumption was categorized by lifetime intake, with individuals considered drinkers if they had consumed alcoholic beverages at least once weekly for a minimum of one year; those not meeting this threshold were classified as non-drinkers [24].

Comorbidities were assessed using the Adult Comorbidity Evaluation-27 (ACE-27) index [25]. Tumors were restaged in accordance with the eighth edition of the AJCC TNM classification system. Based on these TNM values, patients were assigned to stage groups under the eighth edition, and these groupings were subsequently compared with those defined by the seventh edition.

### 2.3. Assessment of HPV Status

HPV status was assessed using p16 immunohistochemistry, a validated surrogate marker for HPV-associated oropharyngeal tumors. Cases were classified as HPV-positive if more than 75% of tumor cells exhibited diffuse nuclear and cytoplasmic staining for the p16 protein [26].

### 2.4. Definitive Treatment

Treatment strategies, surgical or non-surgical, were selected according to National Comprehensive Cancer Network (NCCN) guidelines and recommendations from the multidisciplinary Head and Neck Tumor Board at PUMCH. Individualized decisions also incorporated patient-specific factors such as performance status, comorbid conditions, and personal preferences.

Patients undergoing surgery received primary tumor resection via transoral laser microsurgery (TLM) or open approaches, with neck dissection performed when indicated. Postoperative adjuvant therapy was administered based on established high-risk pathological features, including extracapsular extension (ECE), positive surgical margins, advanced nodal status, or elevated recurrence risk.

For patients managed non-surgically, definitive radiotherapy—with or without concurrent chemotherapy—was delivered in accordance with disease stage and risk stratification, following NCCN recommendations. A subset of patients also underwent induction chemotherapy prior to definitive treatment.

Concurrent chemo-radiotherapy most commonly included cisplatin; however, cetuximab was used selectively in cases where patients were unable to tolerate cisplatin due to toxicity or underlying health conditions. Induction regimens typically involved cisplatin, alone or in combination with paclitaxel, or multi-agent protocols, including cisplatin, docetaxel, and fluorouracil, with or without cetuximab.

### 2.5. Patient Follow-Up and Study Endpoints

Patients underwent clinical and imaging evaluations every three months during the first three years following treatment, every six months during years four and five, and once annually thereafter. Follow-up continued for at least six months or until the occurrence of a predefined clinical event.

The primary endpoint was overall survival (OS), defined as the interval from the date of histologically confirmed OPSCC diagnosis to death from any cause. Secondary endpoints included disease-specific survival (DSS), measured from the start of treatment to death attributable to the disease, and progression-free survival (PFS), defined as the duration during which patients remained alive without evidence of disease progression.

### 2.6. Statistical Analysis

The baseline characteristics of study participants were summarized using descriptive statistics. For continuous variables with a normal distribution, data were reported as means with standard deviations (SDs), while categorical variables were expressed as counts and corresponding percentages.

Survival outcomes, including OS, DSS, and PFS, were estimated using the Kaplan–Meier method, with comparisons between groups performed via the log-rank test.

Independent prognostic indicators were identified using univariate and multivariate Cox proportional hazards regression models. Variables with *p* < 0.10 in univariate analysis were entered into the multivariable model; backward elimination used an entry criterion of *p* < 0.10 and a retention criterion of *p* < 0.15. The proportional hazards assumption was assessed using Schoenfeld residuals. Multicollinearity was evaluated using variance inflation factors (VIFs), prespecifying VIF > 10 as indicating problematic multicollinearity.

Results from the multivariable models were presented as adjusted hazard ratios (aHRs) with 95% confidence intervals (CIs).

An interaction term was incorporated into the multivariate Cox model to assess whether smoking modifies the survival benefit associated with HPV-positive status. All statistical analyses were conducted using R software (version 4.0.2; R Foundation for Statistical Computing, Vienna, Austria). A two-tailed *p*-value of <0.05 was considered indicative of statistical significance.

To assess the robustness of our findings, sensitivity analyses were performed using four models with different covariate adjustment strategies: (1) minimal adjustment (HPV, smoking, interaction only); (2) basic adjustment (adding T and N stages); (3) full adjustment (adding age and sex); and (4) comprehensive adjustment (adding treatment modality).

## 3. Results

### 3.1. Patient Characteristics

This study included 329 patients, with a median age of 59 years (range: 27–94), and a predominance of males (*n* = 254, 77.2%). Positive p16 immunohistochemistry, indicative of HPV-associated disease, was observed in 211 of the 329 cases (64.1%). Based on HPV status, patients were categorized into two groups: 211 (64.1%) were HPV-positive, and 118 (36.9%) were HPV-negative. A total of 181 patients (55%) were classified as smokers, while the remaining 148 (44%) were non-smokers. Detailed demographic and clinical data are presented in Table 1.

No significant differences in adult comorbidity scores were found between the HPV-positive and HPV-negative groups. Compared to those with HPV-negative tumors, patients with HPV-positive OPSCC were more likely to be female (27.5% vs. 14.4%, *p* = 0.007), non-smokers (55.5% vs. 26.3%, *p* < 0.001), and non-drinkers (72.0% vs. 43.2%, *p* < 0.001).

In the HPV-positive subgroup, the most common tumor location was the tonsil (72.5%, *p* < 0.001), and the majority of tumors were classified as AJCC 8th edition stage I or II (52.6% and 33.7%, respectively; *p* < 0.001). Furthermore, most HPV-positive patients received non-surgical definitive treatment (67.8%, *p* = 0.005).

### 3.2. Survival Outcomes Based on HPV Status

The follow-up period for the 329 patients ranged from 6 to 156 months, with a mean duration of 43.2 months and a median of 30 months. To evaluate the prognostic impact of tumor HPV status, survival outcomes were compared between HPV-positive and HPV-negative OPSCC groups.

**Figure 1:**
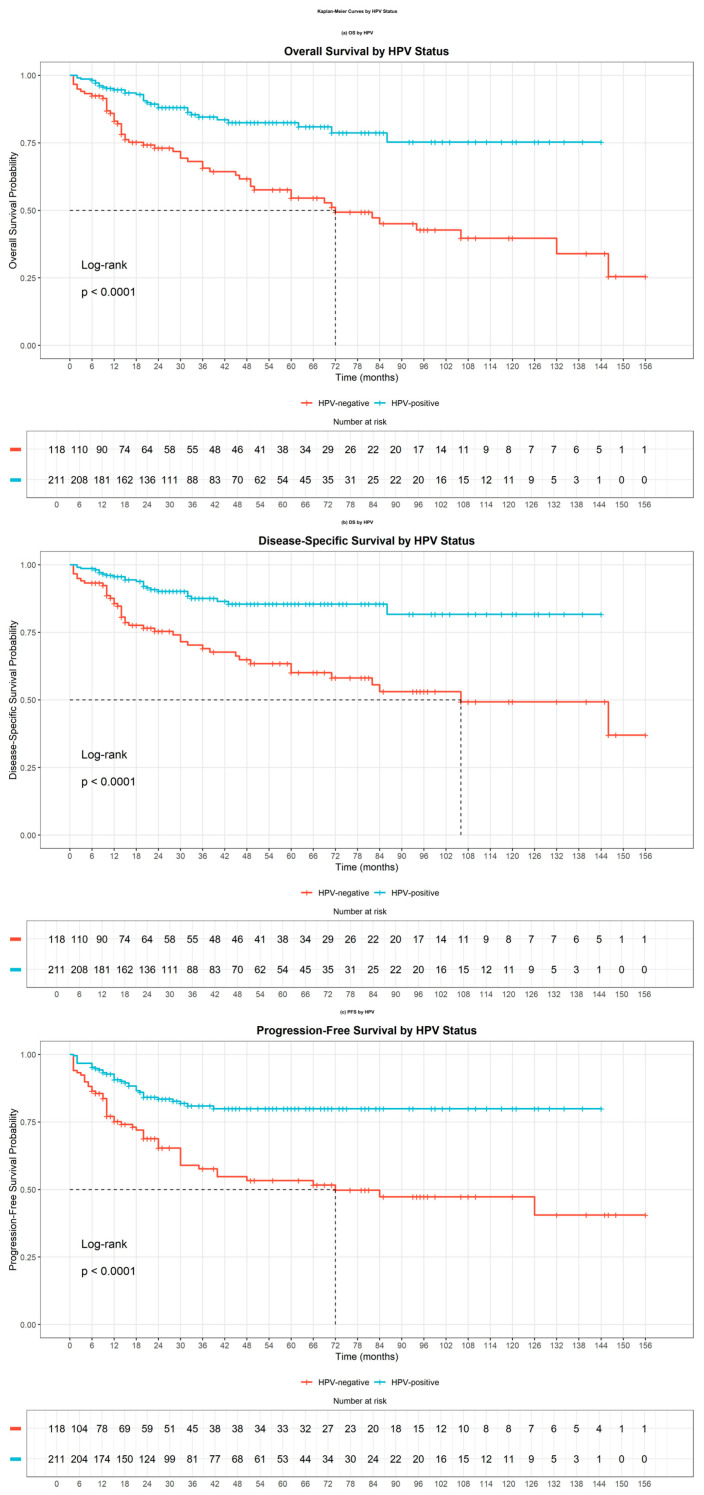
Kaplan–Meier survival curves stratified by HPV status. Panel (**a**) depicts overall survival (OS), showing that HPV-positive patients demonstrated significantly improved survival compared to HPV-negative patients (*p* < 0.05). Panel (**b**) presents disease-specific survival (DSS), which similarly indicates a substantial survival advantage for HPV-positive patients. Panel (**c**) displays progression-free survival (PFS), where HPV-positive status was associated with a reduced risk of disease progression or recurrence. These findings collectively demonstrate that HPV positivity serves as a favorable prognostic biomarker across multiple survival endpoints in this patient cohort.

Kaplan–Meier analysis revealed that patients with HPV-positive OPSCC had significantly improved OS, disease-specific survival (DSS), and progression-free survival (PFS) compared to those with HPV-negative tumors (*p* < 0.001 for all outcomes, log-rank test). Specifically, 3- and 5-year OS rates were 84.5% (95% CI: 77.8–89.3%) and 82.5% (95% CI: 75.2–87.8%) in the HPV-positive group versus 65.6% (95% CI: 55.2–74.1%) and 54.5% (95% CI: 43.4–64.3%) in the HPV-negative group (Figure 1a). Corresponding DSS rates were 87.5% (95% CI: 82.5–92.8%) and 85.4% (95% CI: 79.8–91.4%) for HPV-positive patients, compared to 69.0% (95% CI: 60.2–78.9%) and 60.0% (95% CI: 50.4–71.5%) for HPV-negative patients (Figure 1b). Similarly, PFS rates at 3 and 5 years were 80.9% (95% CI: 75.2–87.1%) and 79.9% (95% CI: 74.0–86.3%) in HPV-positive individuals, in contrast to 57.6% (95% CI: 48.5–68.5%) and 53.3% (95% CI: 43.9–64.7%) in those who were HPV-negative (Figure 1c). These results support the role of HPV positivity as a favorable prognostic indicator across all major survival metrics.

To further explore the influence of smoking, survival was stratified by both HPV and smoking status. Among the cohort, 181 individuals (55.0%) had a history of smoking. Patients were divided into four groups: 117 (35.6%) HPV-positive non-smokers, 94 (28.6%) HPV-positive smokers, 31 (9.4%) HPV-negative non-smokers, and 87 (26.4%) HPV-negative smokers.

**Figure 2:**
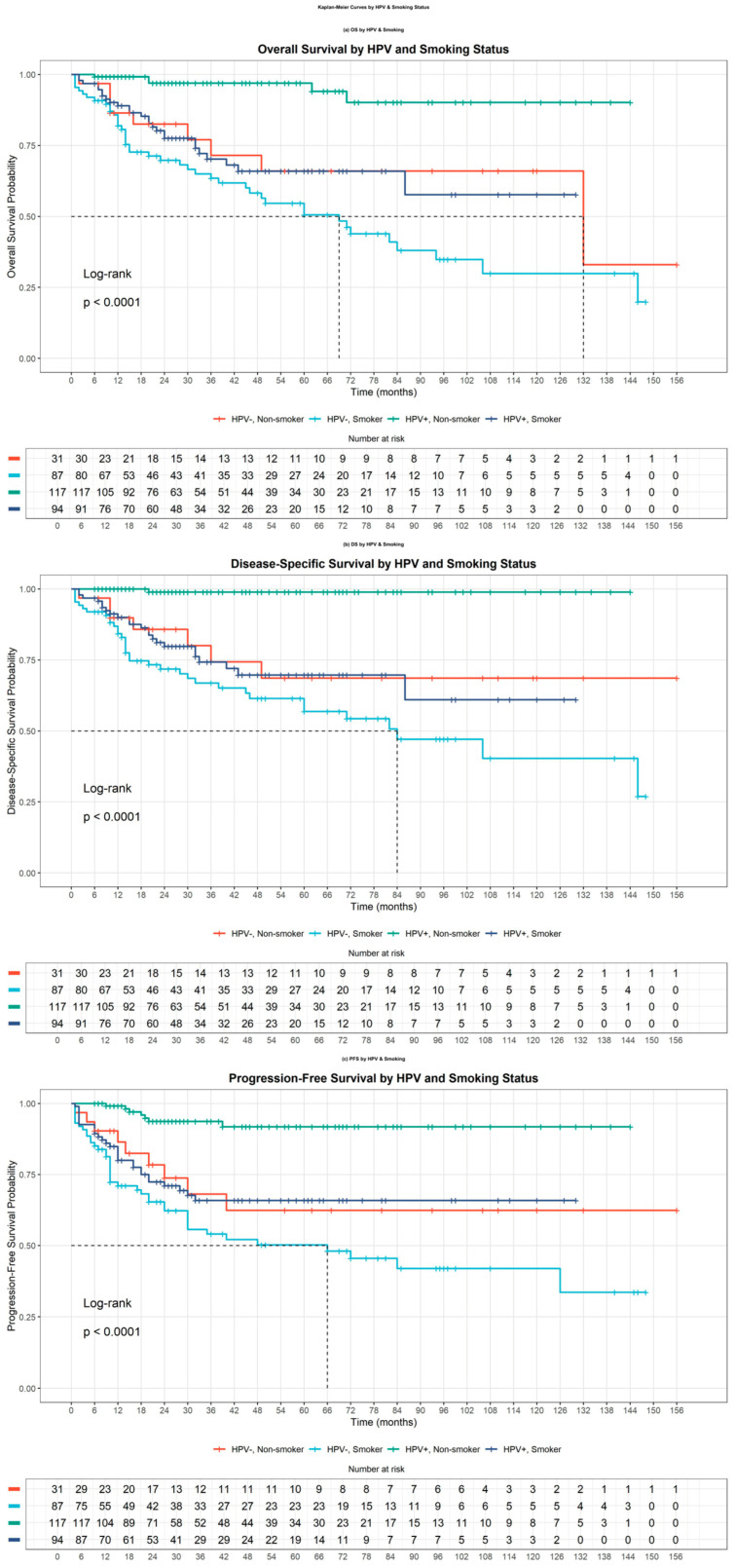
Kaplan–Meier survival analyses with patients stratified by both HPV status and smoking history. Panel (**a**) shows overall survival (OS), revealing that HPV-positive non-smokers exhibited the most favorable outcomes, followed by HPV-positive smokers. In contrast, HPV-negative patients, regardless of smoking history, demonstrated poorer survival rates. Panel (**b**) illustrates disease-specific survival (DSS), maintaining similar stratification patterns, with HPV status appearing to exert a stronger influence than smoking history. Panel (**c**) depicts progression-free survival (PFS), further confirming the dominant impact of HPV status on clinical outcomes while suggesting a potential modifying effect of smoking. The combined analysis indicates that although HPV status remains the primary prognostic factor, smoking history may diminish the survival advantage conferred by HPV positivity.

Kaplan–Meier analysis showed that the most favorable survival outcomes were observed in HPV-positive non-smokers, followed by HPV-positive smokers. Both HPV-negative subgroups—regardless of smoking history—exhibited significantly poorer outcomes (*p* < 0.001 for all comparisons, log-rank test). This stratified analysis indicates that although HPV positivity is a dominant prognostic factor, its associated survival advantage is diminished in patients with a history of smoking (Figure 2).

To explore the joint impact of HPV status and smoking on survival in OPSCC patients, we developed multivariable Cox proportional hazards models with and without interaction terms. The model excluding the interaction showed that HPV positivity significantly reduced mortality risk (HR = 0.531, 95% CI: 0.331–0.853), while smoking was associated with a markedly increased risk (HR = 2.874, 95% CI: 1.567–5.271).

In contrast, the interaction model provided more detailed insights. Among non-smokers, HPV-positive status was strongly protective (HR = 0.148, 95% CI: 0.049–0.443), corresponding to an 85.2% reduction in overall mortality. However, the interaction term (HR = 4.804, 95% CI: 1.439–16.041) revealed that this protective effect was significantly reduced among smokers. When combining the effects (0.148 × 4.804), the resulting hazard ratio was 0.710, suggesting that smoking largely offsets the survival benefit of HPV positivity (Table 2). Compared with HPV-negative non-smokers, HPV-positive non-smokers had an 85% lower risk of mortality, whereas HPV-positive smokers showed no significant survival advantage.

A similar pattern was observed for disease-specific survival. In the basic model, HPV-positive status remained protective (HR = 0.534, 95% CI: 0.315–0.904), while smoking substantially increased disease-specific mortality risk (HR = 4.072, 95% CI: 1.888–8.779). Within the interaction model, HPV-positive non-smokers experienced a pronounced survival benefit (HR = 0.039, 95% CI: 0.005–0.315), indicating a 96.1% risk reduction. However, the interaction term (HR = 19.356, 95% CI: 2.219–168.798) substantially attenuated this benefit in smokers. The combined HR for HPV-positive smokers (0.039 × 19.356 = 0.755) again approximated 1, suggesting the loss of HPV-related survival advantage (Table 3). Compared with HPV-negative non-smokers, HPV-positive non-smokers had a 96% lower risk of disease-specific mortality, whereas HPV-positive smokers showed no meaningful survival advantage.

Progression-free survival showed comparable results. In the absence of interaction, HPV-positive status reduced the risk of disease progression (HR = 0.549, 95% CI: 0.348–0.866), whereas smoking elevated it (HR = 2.720, 95% CI: 1.535–4.820). The interaction model showed that HPV-positive non-smokers had a marked reduction in progression risk (HR = 0.191, 95% CI: 0.071–0.515), but this benefit was diminished by the interaction effect (HR = 3.714, 95% CI: 1.232–11.194). The combined hazard ratio for HPV-positive smokers (0.191 × 3.714 = 0.709) suggested that smoking nullifies the progression-free survival benefit typically associated with HPV positivity (Table 4). Compared with HPV-negative non-smokers, HPV-positive non-smokers had an 81% lower risk of disease progression, whereas HPV-positive smokers showed no significant progression-free survival advantage.

### 3.3. Sensitivity Analysis

Sensitivity analyses confirmed the robustness of the HPV × smoking interaction across different covariate adjustment strategies (Supplementary Table S3). The interaction remained statistically significant across all four adjustment models for overall survival (HR range: 3.52–4.80) and disease-specific survival (HR range: 13.76–19.36), demonstrating that smoking consistently negates the survival benefit of HPV-positive status regardless of the covariates included in the model. For progression-free survival, the interaction showed similar directional effects across most models (HR range: 2.89–3.71), with Model 4 showing a non-significant but consistent trend (HR = 2.89, 95% CI: 0.94–8.88). These findings indicate that the HPV × smoking interaction is a robust phenomenon that is not dependent on specific modeling choices or potential unmeasured confounding.

### 3.4. Interaction Analysis of Survival Outcomes

To explore the joint impact of HPV status and smoking on survival in OPSCC patients, we developed multivariable Cox proportional hazards models with and without interaction terms. The model excluding the interaction showed that HPV positivity significantly reduced mortality risk (HR = 0.531, *p* = 0.00879), while smoking was associated with a markedly increased risk (HR = 2.874, *p* = 0.000647).

In contrast, the interaction model provided more detailed insights. Among non-smokers, HPV-positive status was strongly protective (HR = 0.148, *p* = 0.00064), corresponding to an 85.2% reduction in overall mortality. However, the interaction term (HR = 4.804, *p* = 0.01073) revealed that this protective effect was significantly reduced among smokers. When combining the effects (0.148 × 4.804), the resulting hazard ratio (HR = 0.710) approached 1, indicating that smoking largely offsets the survival benefit conferred by HPV positivity (Table 2). A similar pattern was observed for DSS. In the basic model, HPV-positive status remained protective (HR = 0.534, *p* = 0.01955), while smoking substantially increased disease-specific mortality risk (HR = 4.072, *p* = 0.000342). Within the interaction model, HPV-positive non-smokers experienced a pronounced survival benefit (HR = 0.039, *p* = 0.00237), indicating a 96.1% risk reduction. However, the interaction term (HR = 19.356, *p* = 0.00733) substantially attenuated this benefit in smokers. The combined HR for HPV-positive smokers (0.039 × 19.356 = 0.755) again approximated 1, suggesting the loss of HPV-related survival advantage (Table 3).

PFS showed comparable results. In the absence of interaction, HPV-positive status reduced the risk of disease progression (HR = 0.549, *p* = 0.009973), whereas smoking elevated it (HR = 2.720, *p* = 0.000605). The interaction model showed that HPV-positive non-smokers had a marked reduction in progression risk (HR = 0.191, *p* = 0.00106), but this benefit was diminished by the interaction effect (HR = 3.714, *p* = 0.01976). The combined hazard ratio for HPV-positive smokers (0.191 × 3.714 = 0.709) suggested that smoking nullifies the PFS benefit typically associated with HPV positivity (Table 4).

## 4. Discussion

This study investigated the impact of smoking exposure on survival outcomes among patients with HPV-positive OPSCC, with all cases restaged in accordance with the AJCC 8th edition criteria.

The eighth edition of the AJCC Staging Manual introduced a major revision in the classification of HPV-associated OPSCC, resulting in a notable downstaging of many tumors compared to the previous edition. A substantial proportion of HPV-positive OPSCC cases that were formerly categorized as stage III or IV are now reclassified as stage I or II. This staging shift aligns with the well-documented survival advantage observed in HPV-positive patients relative to those with HPV-negative disease [10].

Historically, tobacco use has been strongly linked to the development of HNSCC, with smokers facing a two- to fourfold higher risk compared to individuals who have never smoked [27,28,29]. Moreover, the risk of HNSCC demonstrates a clear dose–response pattern, increasing with smoking intensity, duration, and cumulative exposure [28]. While this association is well established in HPV-negative HNSCC, comparatively fewer studies have explored the prognostic influence of smoking in HPV-positive OPSCC. Given the rising prevalence of HPV-associated OPSCC, it is crucial to delineate how tobacco exposure affects outcomes in this expanding subgroup of patients. Two meta-analyses, conducted by Chen et al. and Ference et al., demonstrated that smoking negatively influences recurrence and survival in patients with HPV-positive OPSCC, independent of disease stage [30,31]. In contrast, some studies have reported no significant association between tobacco use and HPV-positive HNSCC outcomes [32,33]. These conflicting findings may be partly explained by the inherent limitations of observational research, variations in study methodologies across cohorts, and the failure to clearly differentiate between HPV-positive and HPV-negative HNSCC within the study populations.

The mechanisms by which tobacco use negatively influences prognosis in HPV-positive OPSCC remain unclear. One hypothesis is that tobacco exposure may promote additional genetic alterations, resulting in more aggressive tumor behavior and increased resistance to therapy. Given that nearly two-thirds of individuals with HPV-positive OPSCC are either current or former smokers at the time of diagnosis, this issue deserves deeper investigation [34,35]. Some studies have utilized targeted next-generation sequencing to analyze genes commonly mutated in HNSCC associated with tobacco and alcohol use, comparing HPV-positive OPSCC cases across smoking status. Interestingly, smoking history was not associated with a higher mutation burden in these frequently altered genes, including PIK3CA, MLL2, TP53, FAT1, FBXW7, NOTCH1, and FGFR3. These findings highlight the need for further research to better characterize the molecular profile of HPV-driven OPSCC in the context of tobacco exposure [36].

At present, numerous de-escalation trials are underway to assess the feasibility of reducing chemoradiation intensity or modifying treatment regimens in HPV-associated OPSCC, aiming to minimize treatment-related toxicity while preserving favorable survival outcomes characteristic of this disease compared to HPV-negative cases [37,38,39]. However, not all patients with HPV-positive OPSCC may be suitable candidates for such treatment deintensification. Given the potential for suboptimal therapeutic responses, especially in smokers, smoking status should be carefully evaluated by clinicians prior to initiating de-escalated treatment approaches. Failure to consider this factor may lead to increased risks of treatment resistance and residual disease in HPV-positive smokers.

The influence of smoking on survival outcomes in HPV-positive OPSCC, particularly when adjusted for the AJCC-8 staging system, has not been thoroughly investigated within our national context and warrants further attention. In our analysis, smoking emerged as an independent prognostic factor among HPV-positive OPSCC patients under the AJCC-8 framework. Notably, a history of tobacco use shifted the mortality profile of HPV-positive individuals closer to that of HPV-negative patients, although survival rates in the HPV-positive smoker group remained comparatively higher. These findings are in agreement with previously published systematic reviews.

When stratifying patients by both HPV status and smoking history, the OPSCC cohort was divided into four distinct subgroups. Among these, HPV-positive non-smokers demonstrated the most favorable survival, followed by HPV-positive smokers. Interaction analysis across the subgroups revealed more detailed insights: In non-smokers, HPV positivity significantly reduced mortality risk by 85.2%. However, the presence of a significant interaction term indicated that this survival advantage was substantially weakened in individuals with a smoking history. The calculated combined effect for HPV-positive smokers suggested that the prognostic benefit associated with HPV positivity was nearly nullified in this subgroup.

These findings point to a negative interaction between HPV status and smoking, implying an antagonistic effect on survival outcomes. Additional subgroup analyses stratified by clinical and demographic characteristics further validated the consistency of these results. Taken together, our study underscores the critical need to account for smoking history when evaluating prognosis and formulating treatment strategies for patients with HPV-associated OPSCC. This study is among the first, particularly within an Asian cohort staged uniformly under AJCC-8, to robustly demonstrate through formal interaction analysis that the survival benefit of HPV positivity is effectively nullified in smokers, shifting their risk profile towards that of HPV-negative patients.

This study has several limitations. First, its retrospective design, coupled with reliance on self-reported smoking history, may introduce information and recall bias. Second, as a single-center investigation conducted at a tertiary institution in China, the findings may primarily reflect localized treatment practices and thus limit broader applicability. Expanding this research to a national or international scale through multi-center collaboration would enhance the generalizability and external validity of the results.

In conclusion, our study provides novel evidence from a Chinese cohort staged under AJCC-8, demonstrating a significant negative interaction between HPV status and smoking. Crucially, we show that tobacco use effectively negates the survival advantage typically associated with HPV-positive OPSCC, fundamentally altering risk stratification based solely on HPV status and AJCC-8 stage. This underscores the critical need to integrate smoking history into prognostic models and therapeutic decision-making, especially regarding treatment de-intensification.

## Figures and Tables

**Table 1:** Patient characteristics for HPV-negative and HPV-positive oropharyngeal squamous cell carcinoma patients.

Characteristics		HPV ^a^ Negative (*n* = 118)		HPV ^a^ Positive (*n* = 211)		*p* ^b^ Value
		No. of Patients	%	No. of Patients	%	
Gender						0.007
	Male	101	85.6	153	72.5	
	Female	17	14.4	58	27.5	
Age (years)						0.040
	Mean ± SD	59.75 ± 9.95		57.35 ± 10.28		
Tobacco use						<0.001
	Smoker	87	73.7	94	44.5	
	Non-smoker	31	26.3	117	55.5	
Alcohol consumption						<0.001
	Drinker	67	56.8	59	28.0	
	Non-drinker	51	43.2	152	72.0	
Adult comorbidity score						0.491
	None	62	52.5	126	59.7	
	Mild	25	21.2	44	20.8	
	Moderate	29	24.6	40	19.0	
	Severe	2	1.7	1	0.5	
Tumor site						<0.001
	Tonsil	36	30.5	153	72.5	
	Base of tongue	52	44.1	48	22.7	
	Soft palate, lateral/posterior pharyngeal wall, or NOS ^c^	30	25.4	10	4.7	
AJCC ^d^ clinical stage 8th						<0.001
	I	3	2.5	111	52.6	
	II	23	19.5	71	33.7	
	III	23	19.5	26	12.3	
	IV	69	58.5	3	1.4	
Primary treatment modality						0.005
	Surgical ^e^	57	48.3	68	32.2	
	Non-surgical ^f^	61	51.7	143	67.8	

Abbreviations: ^a^ HPV, human papillomavirus; ^b^ *p*, Pearson’s Chi-squared test; ^c^ NOS, not otherwise specified; ^d^ AJCC, American Joint Committee on Cancer; ^e^ surgical treatment, including surgery only, surgery plus radiotherapy, and surgery plus chemo-radiotherapy; ^f^ non-surgical treatment, including definitive radiotherapy and chemo-radiotherapy.

**Table 2:** Multivariable Cox regression analysis of overall survival.

**A. Without Interaction Terms**							
**Variable**	**β ^b^**	**SE ^c^**	**HR ^d^**	**Lower 95% CL ^e^**	**Upper 95% CL**	***P1***	*p* *
HPV ^a^-positive	−0.633	0.2416	0.531	0.3307	0.8526	0.00879	0.0352
Smoking	1.0556	0.3095	2.874	1.5669	5.2705	0.000647	0.00259
T_new ^f^	0.3273	0.133	1.387	1.069	1.8002	0.01383	0.0553
N_new ^g^	0.319	0.1327	1.376	1.0606	1.7843	0.01623	0.0649
**B. With Interaction Terms**							
HPV-positive	−1.9118	0.56	0.1478	0.04932	0.443	0.00064	0.003202
Smoking	0.2894	0.3763	1.3357	0.63884	2.793	0.44181	0.4418
T_new ^f^	0.3202	0.131	1.3775	1.06564	1.781	0.01447	0.01809
N_new ^g^	0.3554	0.1335	1.4268	1.09839	1.853	0.00774	0.01788
HPV-positive: Smoking	1.5695	0.6151	4.8043	1.43886	16.041	0.01073	0.01788

Abbreviations: ^a^ HPV, human papillomavirus; ^b^ β, beta regression coefficients; ^c^ SE, standard error; ^d^ HR, hazard ratio; ^e^ CL, confidence level; ^f^ T_new, Reclassified T stages per AJCC 8th edition criteria; ^g^ N_new, Reclassified N stages per AJCC 8th edition criteria; * FDR-adjusted *p*-value.

**Table 3:** Multivariable Cox regression analysis of disease-specific survival.

**A. Without Interaction Terms**							
**Variable**	**β ^b^**	**SE ^c^**	**HR ^d^**	**Lower 95% CL ^e^**	**Upper 95% CL**	***P1***	*p* *
HPV ^a^-positive	−0.6278	0.2689	0.5338	0.3151	0.9041	0.01955	**0.02607**
Smoking	1.404	0.392	4.0715	1.8883	8.7787	0.000342	**0.001366**
T_new ^f^	0.4373	0.1483	1.5486	1.158	2.0708	0.003183	**0.006366**
N_new ^g^	0.3019	0.1491	1.3524	1.0098	1.8113	0.04285	**0.04285**
**B. With Interaction Terms**							
HPV-positive	−3.25422	1.07021	0.03868	0.004748	0.3151	0.00237	**0.008615**
Smoking	0.317	0.42332	1.37301	0.59886	3.1478	0.45395	**0.4539**
T_new ^f^	0.42602	0.14565	1.53115	1.15089	2.037	0.00345	**0.008615**
N_new ^g^	0.34551	0.14986	1.4127	1.05314	1.895	0.02114	**0.02642**
HPV-positive: Smoking	2.96298	1.10498	19.35553	2.21943	168.7984	0.00733	**0.01222**

Abbreviations: ^a^ HPV, human papillomavirus; ^b^ β, beta regression coefficients; ^c^ SE, standard error; ^d^ HR, hazard ratio; ^e^ CL, confidence level; ^f^ T_new, Reclassified T stages per AJCC 8th edition criteria; ^g^ N_new, Reclassified N stages per AJCC 8th edition criteria; * FDR-adjusted *p*-value.

**Table 4:** Multivariable Cox regression analysis of progression-free survival.

**A. Without Interaction Terms**							
**Variable**	**β ^b^**	**SE ^c^**	**HR ^d^**	**Lower 95% CL ^e^**	**Upper 95% CL**	***P1***	*p* *
HPV ^a^-positive	−0.6001	0.2329	0.5488	0.3477	0.8662	0.009973	0.009973
Smoking	1.0008	0.2918	2.7203	1.5354	4.8198	0.000605	0.002421
T_new ^f^	0.3375	0.13	1.4015	1.0863	1.808	0.009397	0.009973
N_new ^g^	0.3827	0.1329	1.4662	1.13	1.9024	0.003978	0.007955
**B. With Interaction Terms**							
HPV-positive	−1.6537	0.5049	0.1913	0.07113	0.5147	0.00106	0.005279
Smoking	0.3016	0.3735	1.352	0.65026	2.811	0.41936	0.4194
T_new ^f^	0.3338	0.1283	1.3963	1.0858	1.7956	0.00929	0.01549
N_new ^g^	0.4063	0.1333	1.5012	1.15595	1.9496	0.00231	0.005783
HPV-positive: Smoking	1.3121	0.5629	3.7138	1.23214	11.1939	0.01976	0.02471

Abbreviations: ^a^ HPV, human papillomavirus; ^b^ β, beta regression coefficients; ^c^ SE, standard error; ^d^ HR, hazard ratio; ^e^ CL, confidence level; ^f^ T_new, Reclassified T stages per AJCC 8th edition criteria; ^g^ N_new, Reclassified N stages per AJCC 8th edition criteria; * FDR-adjusted *p*-value.

## Data Availability

Data are contained within the article or Supplementary Material.

## Supplementary Materials

The following supporting information can be downloaded at: https://www.mdpi.com/article/10.3390/jcm14196802/s1, Supplementary Table S1. Test of Proportional Hazards Assumption (Schoenfeld Residuals). Supplementary Table S2. Variance Inflation Factor (VIF) Results. Supplementary Table S3. Multivariable Cox Regression Analysis of Disease-Specific Survival. Supplementary Figure S1. Schoenfeld residual plots for testing the proportional hazards assumption of covariates in the Cox model.

## References

[1] Ang K.K., Harris J., Wheeler R., Weber R., Rosenthal D.I., Nguyen-Tân P.F., Westra W.H., Chung C.H., Jordan R.C., Lu C. (2010). Human papillomavirus and survival of patients with oropharyngeal cancer. N. Engl. J. Med..

[2] Dahlstrom K.R., Calzada G., Hanby J.D., Garden A.S., Glisson B.S., Li G., Roberts D.B., Weber R.S., Sturgis E.M. (2013). An evolution in demographics, treatment, and outcomes of oropharyngeal cancer at a major cancer center: A staging system in need of repair. Cancer.

[3] Gillison M.L., Chaturvedi A.K., Anderson W.F., Fakhry C. (2015). Epidemiology of human papillomavirus-positive head and neck squamous cell carcinoma. J. Clin. Oncol..

[4] Cohen M.A., Weinstein G.S., O’Malley B.W., Feldman M., Quon H. (2011). Transoral robotic surgery and human papillomavirus status: Oncologic results. Head Neck.

[5] Garden A.S., Kies M.S., Morrison W.H., Weber R.S., Frank S.J., Glisson B.S., Gunn G.B., Beadle B.M., Ang K.K., Rosenthal D.I. (2013). Outcomes and patterns of care of patients with locally advanced oropharyngeal carcinoma treated in the early 21st century. Radiat. Oncol..

[6] Elrefaey S., Massaro M.A., Chiocca S., Chiesa F., Ansarin M. (2014). HPV in oropharyngeal cancer: The basics to know in clinical practice. Acta Otorhinolaryngol. Ital..

[7] Fakhry C., Westra W.H., Li S., Cmelak A., Ridge J.A., Pinto H., Forastiere A., Gillison M.L. (2008). Improved survival of patients with human papillomavirus–positive head and neck squamous cell carcinoma in a prospective clinical trial. J. Natl. Cancer Inst..

[8] Kreimer A.R., Johansson M., Waterboer T., Kaaks R., Chang-Claude J., Drogen D., Tjønneland A., Overvad K., Quirós J.R., González C. (2013). Evaluation of human papillomavirus antibodies and risk of subsequent head and neck cancer. J. Clin. Oncol..

[9] Lang Kuhs K.A., Anantharaman D., Waterboer T., Johansson M., Brennan P., Michel A., Willhauck-Fleckenstein M., Purdue M.P., Holcátová I., Ahrens W. (2015). Human papillomavirus 16 E6 antibodies in individuals without diagnosed cancer: A pooled analysis. Cancer Epidemiol. Biomark. Prev..

[10] Lydiatt W.M., Patel S.G., O’Sullivan B., Brandwein M.S., Ridge J.A., Migliacci J.C., Loomis A.M., Shah J.P. (2017). Head and neck cancers-major changes in the American joint committee on cancer eighth edition cancer staging manual. CA Cancer J. Clin..

[11] Colevas A.D., Yom S.S., Pfister D.G., Spencer S., Adelstein D., Adkins D., Brizel D.M., Burtness B., Busse P.M., Caudell J.J. (2018). NCCN Guidelines Insights: Head and Neck Cancers, Version 1.2018. J. Natl. Compr. Cancer Netw..

[12] Lee R.H., Salesky M., Benjamin T., El-Sayed I.H., George J.R., Ha P.K., Ryan W.R., Heaton C.M. (2022). Impact of Smoking and Primary Tumor Subsite on Recurrence in HPV-Associated Oropharyngeal Squamous Cell Carcinoma. Otolaryngol. Head Neck Surg..

[13] Haigentz M., Suarez C., Strojan P., Rodrigo J.P., Rinaldo A., Bradford C.R., Corry J., Takes R.P., Ferlito A. (2018). Understanding interactions of smoking on prognosis of HPV-associated oropharyngeal cancers. Adv. Ther..

[14] Wurdemann N., Wagner S., Sharma S.J., Prigge E.S., Reuschenbach M., Gattenlöhner S., Klussmann J.P., Wittekindt C. (2017). Prognostic impact of AJCC/UICC 8th edition new staging rules in oropharyngeal squamous cell carcinoma. Front. Oncol..

[15] O’Sullivan B., Huang S.H., Su J., Garden A.S., Sturgis E.M., Dahlstrom K., Lee N., Riaz N., Pei X., Koyfman S.A. (2016). Development and validation of a staging system for HPV-related oropharyngeal cancer by the international collaboration on oropharyngeal cancer network for staging (ICON-S): A multicentre cohort study. Lancet Oncol..

[16] Geltzeiler M., Bertolet M., Albergotti W., Gleysteen J., Olson B., Persky M., Gross N., Li R., Andersen P., Kim S. (2018). Staging HPV-related oropharyngeal cancer: Validation of AJCC-8 in a surgical cohort. Oral Oncol..

[17] Nauta I.H., Rietbergen M.M., van Bokhoven A.A.J.D., Bloemena E., Lissenberg-Witte B.I., Heideman D.A.M., Baatenburg de Jong R.J., Brakenhoff R.H., Leemans C.R. (2018). Evaluation of the eighth TNM classification on p16-positive oropharyngeal squamous cell carcinomas in the Netherlands and the importance of additional HPV DNA testing. Ann. Oncol..

[18] Malm I.J., Fan C.J., Yin L.X., Li D.X., Koch W.M., Gourin C.G., Pitman K.T., Richmon J.D., Westra W.H., Kang H. (2017). Evaluation of proposed staging systems for human papillomavirus-related oropharyngeal squamous cell carcinoma. Cancer.

[19] Husain Z.A., Chen T., Corso C.D., Wang Z., Park H., Judson B., Yarbrough W., Deshpande H., Mehra S., Kuo P. (2017). A comparison of prognostic ability of staging systems for human papillomavirus-related oropharyngeal squamous cell carcinoma. JAMA Oncol..

[20] van Gysen K., Stevens M., Guo L., Jayamanne D., Veivers D., Wignall A., Pang L., Guminski A., Lee A., Hruby G. (2019). Validation of the 8(th) edition UICC/AJCC TNM staging system for HPV associated oropharyngeal cancer patients managed with contemporary chemo-radiotherapy. BMC Cancer.

[21] Fakhry C., Westra W.H., Wang S.J., van Zante A., Zhang Y., Rettig E., Yin L.X., Ryan W.R., Ha P.K., Wentz A. (2017). The prognostic role of sex, race, and human papillomavirus in oropharyngeal and nonoropharyngeal head and neck squamous cell cancer. Cancer.

[22] Biotechnology-Biobanking-General Requirements for Biobanking [EB/OL].

[23] Chidambaram S., Nakken E.R., Kennedy W., Thorstad W.L., Chen S.Y., Pipkorn P., Zevallos J.P., Mazul A.L. (2020). Prognostic Significance of Smoking in Human Papillomavirus-Positive Oropharyngeal Cancer Under American Joint Committee on Cancer Eighth Edition Stage. Laryngoscope.

[24] Chen P., Chen X.H., Huang Z.G. (2017). Matched-pair analysis of survival in patients with poorly differentiated versus well-differentiated glottic squamous cell carcinoma. Oncotarget.

[25] Nesic V.S., Petrovic Z.M., Sipetic S.B., Jesic S.D., Soldatovic I.A., Kastratovic D.A. (2012). Comparison of the adult comorbidity evaluation 27 and the charlson comorbidity indices in patients with laryngeal squamous cell carcinoma. J. Laryngol. Otol..

[26] Xiao R., Pham Y., Ward M.C., Houston N., Reddy C.A., Joshi N.P., Greskovich J.F., Woody N.M., Chute D.J., Lamarre E.D. (2020). Impact of active smoking on outcomes in HPV+ oropharyngeal cancer. Head Neck.

[27] Hashibe M., Brennan P., Benhamou S., Castellsague X., Chen C., Curado M.P., Dal Maso L., Daudt A.W., Fabianova E., Fernandez L. (2007). Alcohol drinking in never users of tobacco, cigarette smoking in never drinkers, and the risk of head and neck cancer: Pooled analysis in the International Head and Neck Cancer Epidemiology Consortium. J. Natl. Cancer Inst..

[28] Jethwa A.R., Khariwala S.S. (2017). Tobacco-related carcinogenesis in head and neck cancer. Cancer Metastasis Rev..

[29] Wyss A., Hashibe M., Chuang S.C., Lee Y.C., Zhang Z.F., Yu G.P., Winn D.M., Wei Q., Talamini R., Szeszenia-Dabrowska N. (2013). Cigarette, cigar, and pipe smoking and the risk of head and neck cancers: Pooled analysis in the International Head and Neck Cancer Epidemiology Consortium. Am. J. Epidemiol..

[30] Chen S.Y., Massa S., Mazul A.L., Kallogjeri D., Yaeger L., Jackson R.S., Zevallos J., Pipkorn P. (2020). The association of smoking and outcomes in HPV-positive oropharyngeal cancer: A systematic review. Am. J. Otolaryngol..

[31] Ference R., Liao D., Gao Q., Mehta V. (2020). Impact of smoking on survival outcomes in HPV-related oropharyngeal carcinoma: A meta-analysis. Otolaryngol. Head Neck Surg..

[32] Anantharaman D., Muller D.C., Lagiou P., Ahrens W., Holcátová I., Merletti F., Kjærheim K., Polesel J., Simonato L., Canova C. (2016). Combined effects of smoking and HPV16 in oropharyngeal cancer. Int. J. Epidemiol..

[33] Skoulakis A., Tsea M., Koltsidopoulos P., Lachanas V., Hajiioannou J., Petinaki E., Bizakis J., Skoulakis C. (2020). Do smoking and human papilloma virus have a synergistic role in the development of head and neck cancer? A systematic review and meta-analysis. J. BUON.

[34] Granata R., Miceli R., Orlandi E., Perrone F., Cortelazzi B., Franceschini M., Locati L.D., Bossi P., Bergamini C., Mirabile A. (2012). Tumor stage, human papillomavirus and smoking status affect the survival of patients with oropharyngeal cancer: An Italian validation study. Ann. Oncol..

[35] St Guily J.L., Rousseau A., Baujat B., Périé S., Schultz P., Barry B., Dufour X., Malard O., Pretet J.L., Clavel C. (2017). Oropharyngeal cancer prognosis by tumour HPV status in France: The multicentric papillophar study. Oral Oncol..

[36] Mirghani H., Lacroix L., Rossoni C., Sun R., Aupérin A., Casiraghi O., Villepelet A., Lacave R., Faucher G., Marty V. (2018). Does smoking alter the mutation profile of human papillomavirus-driven head and neck cancers?. Eur. J. Cancer.

[37] Nguyen-Tan P.F., Zhang Q., Ang K.K., Weber R.S., Rosenthal D.I., Soulieres D., Kim H., Silverman C., Raben A., Galloway T.J. (2014). Randomized phase III trial to test accelerated versus standard fractionation in combination with concurrent cisplatin for head and neck carcinomas in the Radiation Therapy Oncology Group 0129 trial: Long-term report of efficacy and toxicity. J. Clin. Oncol..

[38] Gabani P., Lin A.J., Barnes J., Oppelt P., Adkins D.R., Rich J.T., Zevallos J.P., Daly M.D., Gay H.A., Thorstad W.L. (2019). Radiation therapy dose de-escalation compared to standard dose radiation therapy in definitive treatment of HPV-positive oropharyngeal squamous cell carcinoma. Radiother. Oncol..

[39] O’Sullivan B., Huang S.H., Siu L.L., Waldron J., Zhao H., Perez-Ordonez B., Weinreb I., Kim J., Ringash J., Bayley A. (2013). Deintensification candidate subgroups in human papillomavirus-related oropharyngeal cancer according to minimal risk of distant metastasis. J. Clin. Oncol..

