# Supplementary material for: Impact of Smoking on Outcomes in HPV-Positive Oropharyngeal Squamous Cell Carcinoma in a Chinese Cohort Under AJCC 8th Edition Staging
Source: J Clin Med. 2025 Sep 26;14(19):6802. doi: 10.3390/jcm14196802 (PMC12525341; doi:10.3390/jcm14196802)
Supplement: Supplementary file 1 [file jcm-14-06802-s001.zip › jcm-3811778-supplementary.pdf]

Supplementary Table S1. Test of Proportional Hazards Assumption (Schoenfeld Residuals)

Variable	χ^2	df	p-value
HPV	0.0685	1	0.79
Smoking	0.1524	1	0.70
T stage (T_new)	0.5991	1	0.44
N stage (N_new)	0.0445	1	0.83
HPV×Smoking	0.0146	1	0.90
**Global**	1.4402	5	0.92

Supplementary Table S2. Variance Inflation Factor (VIF) Results

Variable	VIF	Interpretation (Threshold = 10)
HPV	5.91	Acceptable (below 10)
Smoking	1.64	No multicollinearity
T_new	1.21	No multicollinearity
N_new	1.21	No multicollinearity
HPV × Smoking	6.57	Acceptable (below 10)

Supplementary Table S3. Multivariable Cox Regression Analysis of Disease-Specific Survival

Variable	β[b]	SE[c]	HR[d]	Lower 95% CL[e]	Upper 95% CL	*p*
**A. Without Interaction Terms**						
HPV[a]-positive	-0.6278	0.2689	0.5338	0.3151	0.9041	0.01955
Smoker	1.404	0.392	4.0715	1.8883	8.7787	0.000342
T_new	0.4373	0.1483	1.5486	1.158	2.0708	0.003183
N_new	0.3019	0.1491	1.3524	1.0098	1.8113	0.04285
**B. With Interaction Terms**						
HPV-positive	-3.25422	1.07021	0.03868	0.004748	0.3151	0.00237
SmokingSmoker	0.317	0.42332	1.37301	0.59886	3.1478	0.45395
T_new	0.42602	0.14565	1.53115	1.15089	2.037	0.00345
N_new	0.34551	0.14986	1.4127	1.05314	1.895	0.02114
HPV-positive: Smoking	2.96298	1.10498	19.35553	2.21943	168.7984	0.00733

Abbreviations: [a], HPV, human papillomavirusm; [b],β, beta regression coefficients; [c], SE, standard error; [d], HR, hazard ratio; [e], CL, confidence level

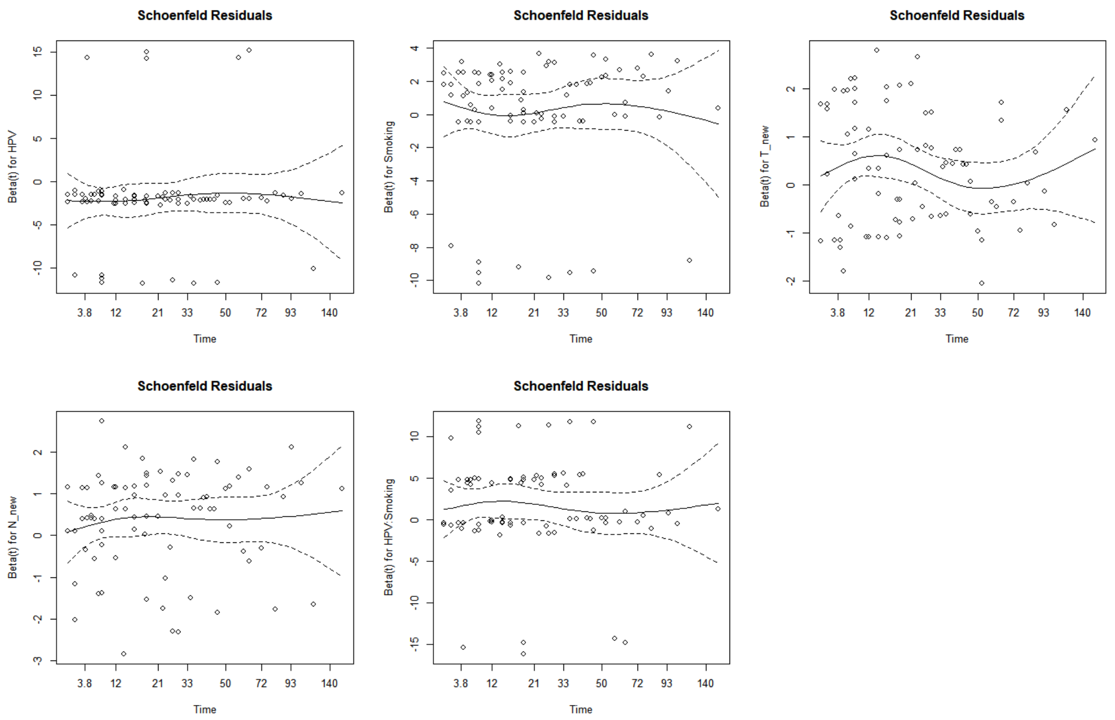

Supplementary Figure S1. Schoenfeld residual plots for testing the proportional hazards assumption of covariates in the Cox model

The proportional hazards assumption was evaluated using Schoenfeld residuals. None of the covariates showed evidence of violation (all $p > 0.05$), and the global test confirmed the assumption was satisfied for the overall model ($\chi^2 = 1.44$, df = 5, $p = 0.92$). Visual inspection of Schoenfeld residual plots further indicated no systematic deviations from zero over time, with fitted smooth curves remaining approximately horizontal within the 95% confidence bands.
